# The Prevalence of Enteric Viruses in Bivalve Molluscs in a Farming Area in Liguria, Northwest Italy

**DOI:** 10.3390/pathogens14010021

**Published:** 2024-12-31

**Authors:** Chiara Masotti, Laura Serracca, Erica Costa, Barbara Betti, Aitor Garcia-Vozmediano, Elisabetta Suffredini, Roberta Battistini

**Affiliations:** 1 Department of Levante Ligure, Istituto Zooprofilattico Sperimentale del Piemonte, Liguria e Valle d’Aosta, Via degli Stagnoni 96, 19100 La Spezia, Italy; chiara.masotti@izsplv.it (C.M.); laura.serracca@izsplv.it (L.S.); 2 Liguria Local Health Unit-ASL 5, Complex Unit of Hygiene of Foods and Animal Origin, 19122 La Spezia, Italy; erica.costa@asl5.liguria.it (E.C.); barbara.betti.veterinaria@asl5.liguria.it (B.B.); 3 Department of Epidemiology—Food Safety, Istituto Zooprofilattico Sperimentale del Piemonte, Liguria e Valle d’Aosta, Via Bologna 148, 10154 Torino, Italy; aitor.garciavozmediano@izsplv.it; 4 Department of Food Safety, Nutrition and Veterinary Public Health, Istituto Superiore di Sanità, 00161 Rome, Italy; elisabetta.suffredini@iss.it

**Keywords:** food safety, norovirus, hepatitis E virus, hepatitis A virus, astrovirus, aichi virus, PCR, shellfish

## Abstract

Bivalve molluscs are filter-feeding organisms, capable of concentrating pathogenic microorganisms from the surrounding environment, thus contributing to the spread of viral pathogens, which they can transmit to humans, especially if eaten raw or undercooked. Although norovirus (NoV) and the hepatitis A virus (HAV) are considered the most common causes of foodborne infections, in recent years, other viruses with a zoonotic potential have been identified in shellfish, such as the hepatitis E virus (HEV), astrovirus (AsV), and aichi virus (AiV). The aim of the study was to investigate the presence of classical and emerging pathogenic enteric viruses in oysters (*Crassostrea gigas*) and mussels (*Mytilus galloprovincialis*) from a mollusc farming area in the northwest of Italy, between April 2022 and March 2023. In the period considered, a total of 168 samples (84 oysters and 84 mussels) were analysed. The prevalence of NoV was highest, with 32.7% (55/168) positive samples, followed by 18.4% (31/168) for AsV and 19.6% (33/168) for AiV. This study revealed, for the first time, the presence of AsV and AiV in molluscs farmed in this sea area. All the samples tested were negative for HAV and HEV. The emergence of new enteric viruses like AiV and AsV in bivalve molluscs underscores the importance of improving surveillance and environmental monitoring methods, particularly in shellfish production areas.

## 1. Introduction

Bivalve molluscs, such as oysters, clams, and mussels, represent a global food resource, providing a primary source of protein for many populations. However, their ability to filter large quantities of water while feeding makes them susceptible to accumulating the pathogens present in the environment, including enteric viruses. Enteric viruses represent a diverse group of pathogens that are responsible for a variety of diseases in humans, and they are transmitted by the faecal–oral route [1]. These viruses pose a significant public health threat due to the fact that they are stable in the environment, they are excreted at high concentrations in the faeces of infected people, and they might reach coastal areas used for shellfish farming through the discharge of either raw (untreated) sewage or inadequately treated wastewater [2]. The transmission of these viruses through the consumption of raw or undercooked shellfish is well documented, with outbreaks regularly occurring worldwide [3]. According to the EFSA 2022 report, norovirus (NoV) (genus *Norovirus*, *Caliciviridae* family) and the hepatitis A virus (HAV) (*Hepatovirus A*, *Picornaviridae* family) are among the enteric viruses that are most often implicated in foodborne outbreaks [4]. In 2021, NoV was the third most frequently reported causative agent in foodborne outbreaks in the EU, with an increase in cases compared to the previous year, although the number was still lower than pre-pandemic levels. This virus was associated with cases reported in 14 Member States and 2 other non-Member States, resulting in a total of 147 human outbreaks, mainly due to the consumption of crustaceans and molluscs and of their products [4]. To date, in Italy, hepatitis A appears to be a predominantly foodborne infection, with 36.8% of cases being due to the consumption of raw or undercooked shellfish [5]. In addition to these "classic" enteric viruses, recently, attention has increasingly focused on emerging viruses that can contaminate bivalve molluscs: among these are the hepatitis E virus (HEV) (*Paslahepevirus balayani, Hepeviridae* family), aichi virus (AiV) (aichivirus A, Picornaviridae family), and astrovirus (AsV) (genus *Mamastrovirus, Astroviridae* family) [6,7,8,9]. These viruses, which are less studied compared to the more well-known enteric viruses, are emerging in new regions and environmental contexts that were previously not identified as high-risk. Environmental pollution and increasing maritime traffic are just some of the factors contributing to the emergence of these new pathogens. The number of reported cases of hepatitis E in Europe showed a progressive increase between 2005 and 2015, reaching 10 times the previous levels. In fact, over 21,000 total cases and a total of 28 deaths have been confirmed, underlining the relevance and the emerging nature of this zoonotic infection [10]. During 2022, in Italy, 44 cases of hepatitis E were reported, diagnosed mostly in the central–northern regions (Emilia-Romagna, Umbria, Abruzzo, and Lombardy). Only 5 cases are attributable to individuals who had stayed in endemic areas (China, Pakistan, and Jordan), while the other 39 (88.6%) are autochthonous cases, to which it was possible to associate, as main risk factors, the consumption of raw or undercooked pork (25.9% of cases) and raw or undercooked wild boar meat (11.5%) [5]. The identification of HEV inside molluscs is rare, and to date, only a few studies have detected the presence of HEV (genotype G3) in molluscs from European production areas [11,12]. Human cases of HEV following shellfish consumption have not yet been clearly demonstrated, although some cases of hepatitis E that were probably linked to shellfish consumption have been described [13,14].

AiV was first identified in an outbreak of acute gastroenteritis in Japan [15]. Though less well known than NoV, AiV is emerging as a potentially food and waterborne pathogen, which may be borne by bivalve molluscs. The virus has been isolated in several geographical regions, suggesting a broader global distribution than initially thought. Recent studies have detected AiV in coastal waters and human faeces, confirming the risk of transmission through the consumption of raw or undercooked seafood [8]. The transmission of this virus, similar to other enteric viruses, primarily occurs through the consumption of food contaminated by wastewater [15]. Although the cases of AiV infection due to shellfish are still under investigation, its epidemiological characteristics make it an emerging virus of interest for food safety.

AsV is another emerging enteric virus associated with human gastroenteritis [16]. Although it is more commonly linked to infections in children, in recent years, it has also been detected in coastal waters and bivalve molluscs [17]. Like NoV, AsV spreads mainly through the consumption of food contaminated by polluted waters. Specifically, these viruses have been isolated in shellfish farming areas, highlighting the risk of transmission, even in the absence of obvious symptoms among the exposed populations [18,19,20,21,22].

Shellfish production areas, especially those near coastal or urban zones, are particularly exposed to contamination by wastewater. The presence of inadequately treated sewage plants or the direct discharge of sewage can serve as a significant source of enteric viruses. Additionally, environmental events like heavy rainfall and flooding can increase the amount of organic pollutants, and consequently pathogens, in shellfish harvesting areas [23]. The monitoring of shellfish production areas plays a crucial role in preventing contamination by both classic and emerging enteric viruses. The constant surveillance of these areas is essential for ensuring food safety and preventing the spread of viral diseases through the consumption of contaminated seafood. The aim of the study was to investigate the presence of classical and emerging pathogenic enteric viruses in Pacific oysters, *Crassostrea* (*Magallana*) *gigas*, and mussels (*Mytilus galloprovincialis*) from important mollusc farming areas in the Ligurian Sea, in the northwestern part of Italy.

## 2. Materials and Methods

### 2.1. Sampling

**Figure 1:**
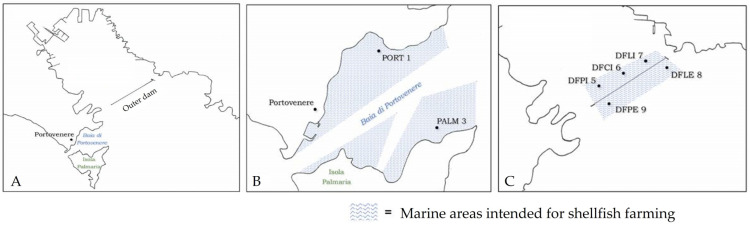
Maps of the study area (**A**) and the details of the sampling point locations (**B**,**C**): PORT 1 (44°3′28.411″ N, 9°50′37.899″ E); PALM 3 (44°3′2.858″ N, 9°51′0.741″ E); DFPI 5 (44°4′21.101″ N, 9°51′31.978″ E); DFCI 6 (44°4′32.61″ N, 9°51′57.409″ E); DFLI7 (44°4′42.352″ N, 9°52′18.278″ E); DFLE 8 (44°4′38.039″ N, 9°52′48.46″ E); DFPE 9 (44°4′12.4″ N, 9°51′45.079″ E).

From April 2022 to March 2023, bivalve molluscs belonging to the two species of *Crassostrea gigas* and *Mytilus galloprovincialis* were sampled every month from 7 points in a class B shellfish farming area, according to EU Reg. 627/2019, in the Ligurian Sea, NW Italy (Figure 1). The shellfish farm is located in front of a highly anthropised coastal area with the presence of a commercial and touristic port and many shipyards. After collection, the samples were transferred to the laboratory in refrigerated conditions and processed immediately. In total, 168 samples (7 points for 12 months for two species) were collected over the study period, of which, 84 were oysters and 84 were mussels. At each sampling, the seawater temperature was registered with a multiparameter sonde (Hydrolab HL7). 

### 2.2. Sample Preparation and Nucleic Acid Extraction

In accordance with ISO 15216-1:2017 [24], for each sample of oysters and mussels, a minimum of 10 individuals were opened, and the hepatopancreas from each animal was carefully dissected. Then hepatopancreases were finely chopped to obtain a paste-like consistency and, subsequently, 2 g of hepatopancreas homogenate was taken. The obtained homogenates were stored at −80 °C before carrying out the analysis. The 2 g hepatopancreas samples were spiked with 10 µL of a Mengovirus (MV) suspension, used as an extraction control, and 2 mL of proteinase K solution (0.1 mg/mL) was added. After vigorous mixing, the samples were incubated at 37 °C with constant shaking for 60 min and then at 60 °C for 15 min in a waterbath. The samples were then centrifuged at 3000× *g* for 5 min and the supernatants were transferred to new vials, after the recording of their volume. Viral RNA extraction was performed using a commercial kit based on magnetic silica and the MiniMag NucliSens platform (bioMerieux, Marcy l’Etoile, France). Additionally, MV RNA was extracted from 10 µL of viral suspension using the same methods and conditions as the test samples, and the RNA obtained was used as a control of the extraction efficiency in the real-time (q)RT-PCR. The extracted nucleic acids were stored at −80 °C until testing.

### 2.3. Real-Time RT-(q)PCR

For the quantitative determination of NoV (genogroups I and II) and HAV, a real-time RT-PCR was performed in accordance with ISO 15216-1:2017 using a C1000 Thermal Cycler (BioRad, Hercules, CA, USA)) and the RNA UltraSense one-step qRT-PCR kit (Invitrogen, Life Technologies, Waltham, MA, USA). The amplification mix contained 1× of UltraSense reaction mix; 500 nM of the forward primer and 900 nM of the reverse primer; 250 nM of the probe; 1.25 µL of RNA Ultrasense enzyme mix; 5 µL of the sample or the control RNA; and molecular grade water to a final volume of 25 µL. The primers and probes used are listed in Table 1. For NoV (GI and GII) and HAV, reverse transcription was performed at 55 °C for 60 min, followed by preheating at 95 °C for 5 min and 45 cycles of PCR (denaturation at 95 °C for 15 s, annealing at 60 °C for 60 s, and extension at 65 °C for 60 s). HEV analyses were performed using the same reaction mix and the same conditions as those of HAV and NoV, with the exception of the reverse transcription, which was performed at 50 °C.

For the qualitative detection of AsV and AiV, the following reaction conditions were used: 1× of Ultrasense reaction mix, 400 nM of both the forward and the reverse primer (Table 1), 200 nM of the probe (Table 1), 1.25 µL of RNA UltraSense enzyme mix, 5 µL of the sample or the control RNA, and molecular grade water to a final volume of 25 µL. Reverse transcription was performed at 50 °C for 30 min, followed by preheating at 95 °C for 5 min and 45 cycles of denaturation at 95 °C for 15 s and annealing–extension at 55 °C or 60 °C for 45 s for AsV and AiV, respectively.

To monitor the correctness of the tests, plasmid RNAs of the various targets were used as a positive control to verify the amplification efficiency and the MV RNA after the virus extraction as a control of the extraction efficiency. Negative PCR controls were added to each run and each target was analysed in duplicate. Amplifications were considered acceptable if the RT-PCR inhibition was ≤75% and the extraction efficiency was ≥1%. A standard curve for NoV GI, GII, HAV, and HEV was used to quantify the amount of the target sequence in each sample; curves with a slope between −3.1 and −3.6 and an R^2^ ≥ 0.98 were considered valid and used for calculations. The number of RNA copies present in each positive sample that could be evaluated was estimated by comparing the sample Ct value to the standard curves. The final concentration was then adjusted, based on the volume of the nucleic acids analysed, and was expressed per gram of hepatopancreas.

The average quantities from the two replicates in each NoV qRT-PCR assay were then calculated to give an overall quantity of the detectable genome copies/g of digestive gland. The limit of the quantification (LOQ) for NoV GI was 140 gc/g, for NoV GII was 130 gc/g, for HAV was 220 cg/g, and finally, for HEV it was 280 cg/g.

### 2.4. Statistical Analysis

The data were managed and analysed using Stata 17 [35]. The overall pathogen-specific prevalences, along with the related binomial exact 95% confidence intervals (CIs), were calculated. Similarly, the occurrence of pathogens was calculated according to the mollusc species under study. Comparisons of the prevalence of the viral agents between the oysters and the mussels were conducted using the Pearson’s Chi-squared test. To gain further insight into the occurrence of NoV, the non-parametric Kruskal–Wallis test was employed to compare the viral loads observed in the molluscan specimens from each collection area. Finally, a linear regression model was constructed to assess the relationship between the NoV loads and the study molluscs, as well as the seasonal temperature of the marine water. This was evaluated separately for each NoV genogroup and for the total (GI + GII). The mean, minimum, and maximum NoV loads were calculated for the oysters and the mussels, separately. All the samples used in the calculations passed quality control criteria for extraction efficiency and RT-PCR inhibition. Values below the LOQ were included in the calculations without modification. For all the statistical analyses, the level of significance was set at *p* < 0.05.

## 3. Results

During the sampling period, NoV was the most frequently detected enteric virus, with a prevalence of positive samples of 32.7% (95% CI = 25.7–40.4; *n* = 55) for GI or GII. A similar prevalence was observed for AsV, which displayed a prevalence of 18.5% (95% CI = 12.9–25.2), and for AiV, for which we recorded a 19.6 % (95% CI = 13.9–26.5; *n*= 33) prevalence. All the samples examined were negative for HAV and HEV (Table 2).

Overall, 38.7% (95% CI = 31.3–46.5; *n* = 65) of the samples analysed were positive for at least one viral target. The percentage of samples contaminated by more than one virus was 25.0% (95% CI = 18.7–32.3; *n* = 42), and of these, 11 samples were positive for NoV GI, NoV GII, AsV, and AiV together. Furthermore, in 12 samples that were positive for NoV GI and GII, positivity was also detected for AsV, while in 17 samples, the simultaneous presence of NoV GI, GII, and AiV was detected. Finally, nine samples were positive only for the two genogroups of NoV, and two samples were positive only for AiV and AsV, but not for NoV overall. NoV was found in 34.5% (95% CI = 24.5–45.7; *n* = 29) of the oysters and 31.0% (95% CI = 21.3–42.0; *n* = 26) of the mussels analysed.

**Figure 2:**
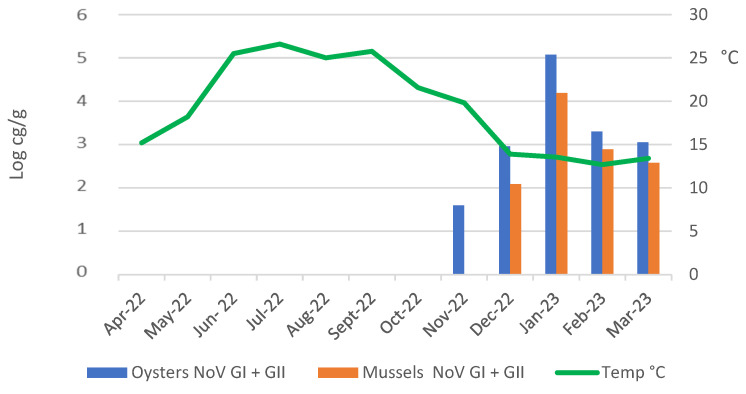
NoV concentration in oysters and in mussels during one year of monitoring.

Concerning NoV detection, the positive samples, both of the mussels and the oysters, were concentrated in the months between December and March, while in the remaining months, only one sample was found to be positive, in November. The occurrence of NoV was comparable in the mussels and the oysters (Pearsons’ Chi-squared test, *p* = 0.622); however, differences in the viral load (the sum of NoV GI and GII) were observed (*p* = 0.003). In particular, the oysters displayed a higher NoV load (mean = 2.97 × 10^4^; min.-max = 39.5–1.8 × 10^5^), compared with the mussels (mean = 4.5 × 10^3^; min.-max = 10.8–3.5× 10^4^). Moreover, the maximum value of NoV concentration was registered in January (*p* < 0.001) (11.8 × 10^4^ genome copies (cg)/g were recorded in the oysters and 15.4 × 10^3^ cg/g in the mussels), when all the samples analysed were positive (Table 2 and Figure 2). Furthermore, NoV was detected in samples from all the collection areas, with no significant differences between the areas.

**Figure 3:**
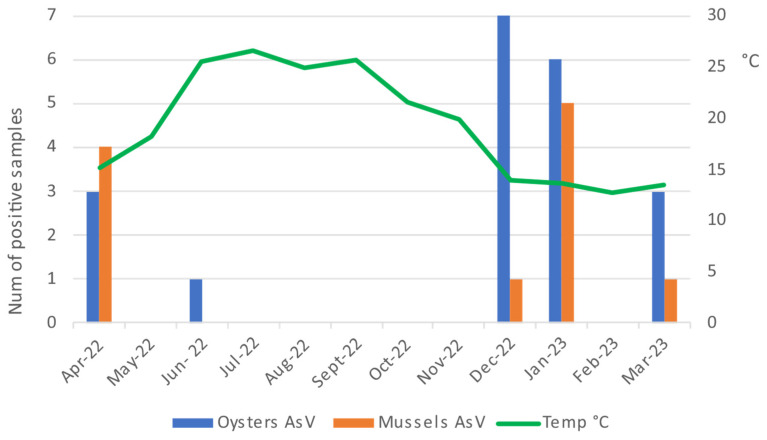
Number of positive samples for AsV in oysters and mussels during one year of monitoring.

AsV RNA was found in 13.1% (95% CI = 6.7–22.2; *n* = 11) of the mussel samples and 23.8% (95% CI = 15.2–34.3; *n* = 20) of the oyster samples. No differences in occurrence were found according to the mollusc species (Pearson’s Chi-squared test, *p* = 0.073). Most of the positive samples for AsV were detected in the months of December and January, and they were more sporadic in the months of March, April, and June. The negative samples were concentrated in the summer months, in particular from July to November in the oysters and from May to November in the mussels (Figure 3).

**Figure 4:**
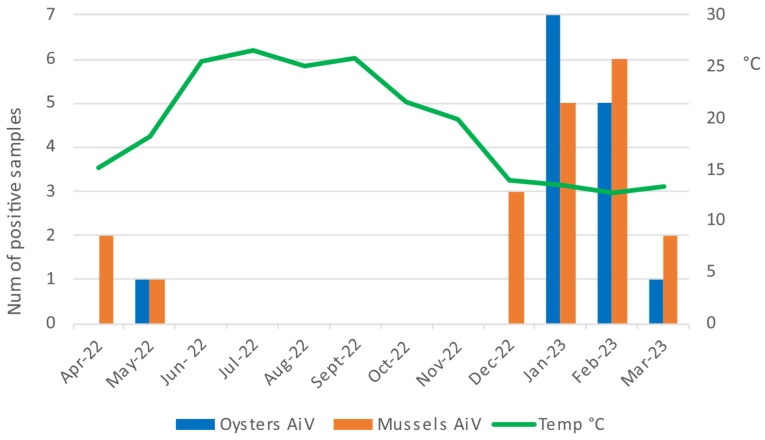
Number of positive samples for AiV in oysters and mussels during one year of monitoring.

Unlike AsV, AiV RNA was found in a higher percentage in the mussels, 22.6% (95% CI = 14.2–33.0; *n* = 19), compared to the oysters, in which we found a prevalence of 16.7% (95% CI = 9.4–26.4; *n* = 14). However, the difference was not statistically different (Pearson’s Chi-squared test, *p*= 0.332). Also, for this virus, positive samples were detected mostly in the winter months and in smaller numbers in spring, while from June to November, no positivity was found (Figure 4).

## 4. Discussion

The viral contamination of the bivalve molluscs found in this study highlights a possible food safety concern in relation to the consumption of these raw or undercooked products, since more than a third of the samples tested positive for at least one viral target and, especially during the entire winter period, the presence of enteric viruses was relevant and consistent. Overall, the data obtained in this study may be indicative of the continuous release of viruses into the marine ecosystem as they are eliminated by the population in the investigated area. In fact, the shellfish farm is located in front of a highly anthropised coastal area, where the main causes of disturbance are coastal urban development and port activities that can cause the presence of several polluted discharges in the area. NoV was the most frequent virus, with a prevalence of 32.7%, in accordance with its known role as the main cause of food poisoning related to the consumption of molluscs [4]. According to the data available in the literature, the prevalence of NoV in bivalve molluscs at different shellfish production levels varies from 0% to 95.6%, depending on the country considered and with significant variations also existing between the different countries within a continent [36,37]. Our data are in line with the values recorded in the production areas of the Mediterranean basin. Similar values (40%) were also found in the regions of the Iberian Peninsula along the coasts of the Atlantic Ocean, as well as in Portugal, 37% [20,37,38,39,40]. In the present study, NoV was found mainly in the winter months, confirming that which was reported in previously studies [40,41]. The statistical analysis also revealed no significant differences in the NoV prevalence or the viral loads between the collection areas. This finding may be attributed to the fact that the areas are in close proximity to each other, with very little difference in the water temperature and being equally affected by pollutants from the coastal zone. Temperature is a known environmental factor that influences viral persistence; its uniformity likely minimized the variation between the areas. The comparison between the viral loads of NoV in the two bivalve mollusc species showed a higher value in the oysters than in the mussels by a factor of 10, which resulted in statistical significance, in agreement with the scientific literature [40]. Oysters pose a greater risk than mussels since they are generally consumed raw. It is important to remember that to reduce the risk of contamination, a heat treatment is required that reaches a temperature of at least 90 °C for 90 s in the internal pulp of the mollusc [42]. Regarding the frequency of the two genogroups (GI and GII), no substantial difference was found, although in the literature this varies greatly based on the sampling sites or the years [20,38]. Instead, in the samples examined, the viral load of GII was higher than that of GI, both in the oysters and the mussels [40,41,43]. The most likely explanation for these data is that GII NoV strains have a high prevalence in the community and that this greater presence translates into a higher viral load in the sewage. Further to this, other studies underline that the difference in bioaccumulation is due to the presence of specific ligands in the different systems of molluscs [44]. Regarding the quantitative levels of NoV detected, it is important to underline that PCR detects the presence of viral RNA, which can derive from both viable and non-viable, or lysed, viruses. Therefore, the prevalence and quantity data reported may reflect the contamination of samples with faecal material contaminated with NoV, but do not provide information on the actual infectivity of the samples. However, several studies have shown that the amount of NoV in the oysters determined by the PCR methods is related to the probability of association with human disease; therefore, the results obtained by the RT-PCR are to be considered relevant for public health [45,46,47], demonstrating that as the level of genomic copies/gram increases, the risk of foodborne infection increases. According to previous study, NoV values above 500 genomic copies indicate a high risk of infection and epidemic [48]. In support of these hypotheses, according to a recent report, EFSA has, therefore, highlighted the need to establish quantitative limits in shellfish, in particular for the oyster/NoV combination, in relation to the prevalence and loads of this virus. As this limit should apply to all EU states, it is possible that this threshold value adapts to the average NoV concentration observed in Europe in production areas [45,46,49]. This value could probably be lower compared to the level of NoV that was detected in this study, especially for oysters in the coldest period of the year. In the near future, therefore, if such a microbiological criterion for shellfish is to be defined, this could be burdensome for local producers who may have the possibility to sell the product only in certain periods of the year, resulting in high economic losses. However, it could represent an opportunity to improve management strategies for shellfish production. The use of the quantitative RT-PCR method, unlike the qualitative method, is important to gain a clear picture of the current situation at the local level and to understand how to improve the future prospects of the sector, ensuring a safer product for consumers.

In this study, AsV and AiV were found in shellfish with prevalences of 18.4% and 19.6%, respectively. According to the available literature, this is the first evidence of the presence of these viruses in shellfish farmed in the Ligurian Sea in the NW of Italy. Available studies on AsV in bivalve molluscs have detected the virus at a detection rate ranging from 6 to 61% [50,51]. In southern Italy, AsV has been reported, with percentages ranging from a minimum of 14.2% to a maximum of 32%, depending on the years studied [18,20]. Similarly, AiV has been found in southern Italy, with a prevalence ranging from 1.8% [52] to 13.25% [18], depending on the area and the year analysed. The variability of the AiV occurrence in these studies appears to have been influenced by weather conditions, as it was lower in the driest years and higher with increased rainfalls. The presence of AiV in other countries varies from 6% in Spain [53] and Tunisia [54] to 8% in France [7]. Contrary to what was reported in a French study [19], in our study, AsV was found in higher numbers in the oysters sampled than in the mussels; the seasonal distribution of the virus, however, reflected the trend therein reported. In our study, the prevalence of AiV was higher in the mussels than in the oysters, hence displaying a distribution opposite to AsV. However, for these two viruses, the difference in prevalence between the species was actually not statistically significant. This indicates that these viruses accumulate similarly between the two species. Such uniformity may reflect the absence of potential species-specific factors that could influence the accumulation or retention of the two viruses in one species, compared to the other, as occurs for NoV. Our study, although in line with the rare studies present in the literature, needs multi-year monitoring to better investigate the trend and the prevalence of AsV and AiV. Regarding the possible effect of purification on the viral load of these two emerging viruses, no studies are reported in the literature. Presumably, since their presence has been demonstrated in purified bivalve molluscs intended for marketing, it can be assumed that it is not effective, as already demonstrated for other enteric viruses [55]. To better understand the efficacy of purification on these two viruses, further studies should be conducted to evaluate the viral load before and after purification. Although the consumption of molluscs has been identified as a possible risk factor for the transmission of foodborne HEV in humans, in our study, all the samples were negative for HEV. These data confirm a study carried out in 2018–2019, lasting one year, in the same area [40]. In central and southern Italy, conversely, the presence of HEV was reported in farmed molluscs, with prevalences of 9.41% and 2.6%, respectively [11,56]. Likewise, no positive samples for HAV were found in the molluscs analysed in our study. In the various studies carried out in Italy, the prevalence of HAV varies from 0.9% to 3.8%, but is mainly concentrated in the regions of southern Italy [57].

## 5. Conclusions

In conclusion, the results from this study confirmed the presence of enteric viruses, pathogenic for humans, in molluscs. Furthermore, the evidence on the occurrence of viruses like AiV and AstV in bivalve molluscs underscores the importance of improving surveillance and environmental monitoring methods, particularly in shellfish production areas. The ability of enteric viruses to contaminate coastal waters and persist in the environment makes them particularly challenging to detect and control. Furthermore, the resilience of these viruses to adverse environmental conditions and traditional shellfish purification methods emphasises the need to develop new mitigation strategies to prevent outbreaks and ensure the safety of seafood consumption on a global scale.

## Figures and Tables

**Table 1:** Primers and probes used in this study for the real-time RT-PCR analysis of each target.

Virus	Primers, Probes	SEQUENCE (5′–3′)	Ref.
MV	MENGO 110	GCGGGTCCTGCCGAAAGT	[25]
	MENGO 209	GAAGTAACATATAGACAGACGCACAC	
	MENGO 147	FAM-ATCACATTACTGGCCGAAGC–MGB	
HAV	HAV68	TCACCGCCGTTTGCCTAG	[26]
	HAV240	GGAGAGCCCTGGAAGAAAG	
	HAV150	FAM-CCTGAACCTGCAGGAATTAA–MGBNFQ	
NoV GI	QNIF4	CGCTGGATGCGNTTCCAT	[27,28]
	NV1LCR	CCTTAGACGCCATCATCATTTAC	
	NVGG1p	FAM-TGGACAGGAGAYCGCRATCT-TAMRA	
NoV GII	QNIF2	ATGTTCAGRTGGATGAGRTTCTCWGA	[29,30]
	COG2R	TCGACGCCATCTTCATTCACA	
	QNIFS	FAM-AGCACGTGGGAGGGCGATCG-TAMRA	
HEV	JVHEV-F	GGTGGTTTCTGGGGTGAC	[31,32]
	JVHEV-R	AGGGGTTGGTTGGATGAA	
	JVHEV- Pmod	FAM-TGATTCTCAGCCCTTCGC–MGB	
AsV	AV1	CCGAGTAGGATCGAGGGT	[33]
	AV2	GCTTCTGATTAAATCAATTTTAA	
	AVs	FAM-CTTTTCTGTCTCTGTTTAGATTATTTTAATCACC-TAMRA	
AiV	AiV-AB-F	GTCTCCACHGACACYAAYTGGAC	[34]
	AiV-AB-R	GTTGTAACATRGCAGCCCAGG	
	AiV-AB-TP	FAM-TTYTCCTTYGTGCGTGC-MGBNFQ	

**Table 2:** The results of the enteric viruses’ RNA presence in the samples for each month during the testing period.

Month	Number of Positive Samples/Number of Samples Analysed (%)					
	HAV	NoV GI	NoVGII	HEV	AsV	AiV
Apr.	0/14 (0.0)	0/14 (0.0)	0/14 (0.0)	0/14 (0.0)	7/14 (50.0)	2/14 (14.3)
May	0/14 (0.0)	0/14 (0.0)	0/14 (0.0)	0/14 (0.0)	0/14 (0.0)	2/14 (14.3)
Jun.	0/14 (0.0)	0/14 (0.0)	0/14 (0.0)	0/14 (0.0)	1/14 (7.1)	0/14 (0.0)
Jul.	0/14 (0.0)	0/14 (0.0)	0/14 (0.0)	0/14 (0.0)	0/14 (0.0)	0/14 (0.0)
Aug.	0/14 (0.0)	0/14 (0.0)	0/14 (0.0)	0/14 (0.0)	0/14 (0.0)	0/14 (0.0)
Sept.	0/14 (0.0)	0/14 (0.0)	0/14 (0.0)	0/14 (0.0)	0/14 (0.0)	0/14 (0.0)
Oct.	0/14 (0.0)	0/14 (0.0)	0/14 (0.0)	0/14 (0.0)	0/14 (0.0)	0/14 (0.0)
Nov.	0/14 (0.0)	1/14 (7.1)	0/14 (0.0)	0/14 (0.0)	0/14 (0.0)	0/14 (0.0)
Dec.	0/14 (0.0)	12/14 (85.7)	10/14 (71.4)	0/14 (0.0)	8/14 (57.1)	3/14 (21.4)
Jan.	0/14 (0.0)	14/14 (100.0)	14/14 (100.0)	0/14 (0.0)	11/14 (78.6)	12/14 (85.7)
Feb.	0/14 (0.0)	13/14 (92.9)	14/14 (100.0)	0/14 (0.0)	0/14 (0.0)	11/14 (78.6)
Mar.	0/14 (0.0)	12/14 (85.7)	14/14 (100.0)	0/14 (0.0)	4/14 (28.6)	3/14 (21.4)
Total	0/168 (0.0)	52/168 (31.0)	52/168 (31.0)	0/168 (0.0)	31/168 (18.4)	33/168 (19.6)

## Data Availability

All the data supporting the present study are reported in this study. Further inquiries can be directed to the corresponding author.
